# Men Who Suffered Intimate Partner Violence: Impressions About Existing Public Campaigns and Recommendations for New Ones

**DOI:** 10.1177/08862605221108075

**Published:** 2022-08-03

**Authors:** Eduardo Reis, Patrícia Arriaga, Carla Moleiro

**Affiliations:** 1 CIS-ISCTE/ISCTE-Instituto Universitário de Lisboa, Lisbon, Portugal

**Keywords:** intimate partner violence, men, campaigns, formative evaluation research, social marketing

## Abstract

Over the last decades, the negative effects of intimate partner violence (IPV) directed at men in abusive different-sex and same-sex relationships have been increasingly investigated. Men who are the targets of IPV face many barriers to help-seeking, and to overcome them, public awareness campaigns have been developed. Women who experienced IPV have found campaigns targeting them to be harmful and misleading, and previous research suggests that following the principles of formative evaluation research may improve campaigns’ effectiveness and reduce unwanted negative effects. This article documents the theory-based formative evaluation research conducted with 14 men abused in different-sex and same-sex relationships for the creation of targeted campaigns. Through semi-structured interviews, men were asked about their overall knowledge of campaigns, their thoughts about specific pictorial IPV campaigns, and their suggestions for the development of new campaigns. Thematic analysis and a theoretically grounded coding scheme were used to analyze the content of the interviews with high interrater reliability. Overall, our results indicate that most men were not aware of campaigns in Portugal, and their impressions about the ones they recalled were mixed. Most men praised clear messages informing forms of violence, while some responded negatively to the inclusion of words such as “shame” and “victim” and the depiction of bruises. They also considered that future campaigns targeting men should portray “real people” like them and provide information on self-efficacy, the efficacy of recommended responses, and threat susceptibility. Our findings are consistent with previous evidence with women who experienced IPV but also provide theoretically grounded novel contributions and highlight the importance of considering the population of interest’s insights when developing and testing new campaigns.

## Introduction

The purpose of this study is to understand the impressions and needs of 14 Portuguese men who were abused in different-sex and same-sex relationships based on theory-based formative evaluation research. By using an explorative and qualitative approach, the results of this formative evaluation research can inform the development of campaigns targeted at these men. This study’s literature review firstly elaborates on intimate partner violence (IPV) and its effects on men in different-sex and same-sex relationships as well as evidence on previously developed IPV campaigns. Subsequently, the tenets of social marketing (SM) and formative evaluation research are also elaborated upon. Lastly, we describe two theoretical models that illustrate different key components that public awareness campaigns can include, as well as their possible effects on target audiences.

IPV has been a subject of study for decades, as a phenomenon that spans across the globe and significantly impacts the health of millions of people every year (Heise & García-Moreno, 2012). It is defined as the victimization of someone by a person with whom he/she has or had an intimate relationship, leading to short or long-term negative health outcomes on a physical (e.g., slapping, hitting), psychological (e.g., intimidation, humiliation), and/or sexual (e.g., sexual coercion) level (Heise & García-Moreno, 2012). IPV can be experienced and perpetrated by both men and women, independently of age, marital status, or sexual orientation (Ali et al., 2016). Thus far, research on IPV has mainly focused on victimized women in different-sex relationships. Evidence suggests that when compared with victimized men, women experience more frequent and severe violence that results in long-lasting impacts on their health (Hamberger & Larsen, 2015).

Nonetheless, an ever-growing body of research has highlighted how men in different-sex and same-sex relationships are the targets of IPV, and how they experience specific processes and victimization types (Edwards et al., 2015; Nowinski & Bowen, 2012). In terms of prevalence, it has been estimated that one in four men experiences some form of IPV in their lifetimes (Nowinski & Bowen, 2012). In 2020, as reported by the Ministry of Internal Affairs (2021), IPV was the most commonly reported crime in Portugal, representing 7.8% of general criminality (*n* = 23,439). Out of these reports, 25% referred to men who had been victimized (*n* = 6,909) (Ministry of Internal Affairs, 2021). For men in same-sex relationships, victimization rates seem to be comparable or even greater than for men in different-sex relationships (Nowinski & Bowen, 2012). This may be due to different minority stressors that lesbian, gay, bisexual, and trans people (LGBT) may experience, such as perceived and actual discrimination, which is also common in IPV and may enhance existing vulnerabilities for LGBT people in abusive relationships (Stiles-Shields & Carroll, 2015).

Help-seeking rates have also been found to be different across populations. In Portugal, the National Support Network for Victims of Domestic Violence assisted 66 men in 2021, when compared with 2,877 women in the same year (Commission for Citizenship and Gender Equality, n.d.-a). Evidence suggests that gay men may seek help at higher rates than heterosexual males (Edwards et al., 2015). To facilitate help-seeking and provide crucial information for the early recognition of signs of abuse, IPV awareness can be achieved through public campaigns. The use of well-developed public awareness campaigns has been found to result in small-to-moderate effects on knowledge, beliefs, attitudes, and some pro-health behaviors (e.g., drug abuse, seatbelt use, oral health) (Noar, 2006). In the context of IPV, evidence on the effectiveness of public awareness campaigns in changing behaviors is limited, but some changes have been found in knowledge and attitudes about IPV (Harvey et al., 2007). Nevertheless, these campaigns have been posited as one of the possible strategies to reach IPV survivors that should be implemented in conjunction with other efforts (e.g., community-based prevention) (Harvey et al., 2007).

### IPV Campaigns

Research in the field of IPV campaigns often focuses on adult females who experience violence, employing mostly qualitative methods to understand the characteristics of campaigns and their potential impact on female targets of violence (Martín et al., 2019). In this line of research, West (2013) has found that abused women considered IPV campaigns to often be emotionally harmful, inaccurate, and misleading, by, for example, depicting severe and graphic aggression. Magaraggia and Cherubini (2017) also highlighted that graphic depictions of physical aggression have become so commonplace that they can inadvertently contribute to the invisibility of other types of violence, such as psychological aggression.

Quantitative evidence is also mixed regarding campaigns’ effectiveness on health outcomes. In their review of public campaigns on IPV, Campbell and Manganello (2013) found some support for changes in attitudes about IPV, facilitation of bystander behavior, and helpline calls in the general population. Nonetheless, as assessed by the study’s authors, the low methodological quality of some studies limited the extrapolations about campaign effectiveness (Campbell & Manganello, 2013). Contrasting with these approaches that focused exclusively on females, Shortland and Palasinski (2019) used a within-subjects design to expose a sample of males and females to 12 posters: six depicting victimized males and six representing victimized females. After exposure, participants quantitatively rated how effective each poster was, and no assessment of previous victimization was conducted. When considering their global sample (i.e., the impressions of both males and females in their sample), the authors found that posters presenting females who were the targets of IPV were overall seen as more effective than posters representing male targets of violence. Furthermore, a systematic content analysis of IPV pictorial campaigns directed at men in different-sex and same-sex relationships found that many campaigns integrated constructs of different theoretical models but often did not articulate them as recommended (Reis et al., 2020). This could impact their effectiveness and ultimately lead to unwanted negative cognitive consequences for the targets of the message, such as denial, defensive avoidance, and/or reactance to message content (i.e., a motivation to resist a threat to freedom) (Shen & Coles, 2015; Witte et al., 2001).

Studies addressing the impact of campaigns oriented to victimized males or assessing victimized males’ impressions of campaigns targeted at them are extremely scarce. This is reflected by the numerous recommendations that have been made on the need for IPV awareness efforts that specifically inform male targets of violence about what constitutes violence, which resources are available, and what information they provide (Hine et al., 2020; Martín et al., 2019). In Portugal, most widespread IPV campaigns have been promoted by the Portuguese Government’s Commission for Citizenship and Gender Equality (CiG) and by the Portuguese Association for Victim Support (APAV). In this country, very few campaigns portray IPV against males, and when they do, men are usually portrayed in conjunction with victimized women (Commission for Citizenship and Gender Equality, n.d.-b). To the best of our knowledge, only one IPV campaign in Portugal thus far has been created exclusively for victimized males (i.e., its sole focus is victimized males, and the campaign’s template is not simultaneously used to target other populations such as victimized females) (APAV, n.d.). Moreover, no peer-reviewed research is available on the impressions or effectiveness of Portuguese campaigns directed at victimized males. Very few studies in this field have documented how these campaigns were created, and if so, according to which standards and guidelines (Donovan & Vlais, 2005).

### SM and Formative Evaluation Research

The principles of SM are an example of key theoretical guidelines in the development of campaigns. SM is defined as a set of procedures that makes use of marketing principles and techniques to elicit behavior change in a given target audience, potentially benefiting society as well (Lee & Kotler, 2019). Harvey et al. (2007) indicate that campaigns based on SM have higher odds of being effective in changing knowledge, attitudes, and social norms about IPV when compared to traditional public education campaigns that do not resort to this methodology. Research on the development of SM campaigns and effective interventions highlights the central role of formative evaluation research and its potential beneficial impact on domestic violence (Lee & Kotler, 2019).

Formative evaluation research is the process through which the target audience’s characteristics, predispositions, and evaluations are collected before implementing the campaign, thus representing foundational research that supports subsequent efforts (Rice & Atkin, 2013). The importance of formative research evaluation is highlighted by the specificities that different types of messages and channels have, since communicating via static images, audio, and video formats entails different conceptualization, implementation, and evaluation strategies (Noar, 2006). Using this research approach may lead campaign designers to understand how susceptible and severe an audience may feel toward a given health threat and also provides information on how to engage their attention, how to overcome audience resistance to a given message, and how counterproductive effects may arise (Rice & Atkin, 2013). Data derived from the implementation of this approach can improve messages during their creation, which is crucial given that most campaigns do not follow a systematic approach and may fail to meet their goals to positively change knowledge, attitudes, and behaviors (Rice & Atkin, 2013).

Different researchers have considered the insights of men (Donovan et al., 1999; Stanley et al., 2009; Thomson et al., 2013) to document the development of campaigns targeting male perpetrators of IPV, but the inquired participants were either from the general population or from perpetrators of violence. Thus far, no studies have assessed the opinions of victimized men in different-sex and same-sex relationships to inform the development of campaigns targeted at victimized men. Moreover, when developing public campaigns, the importance of considering theoretical models to potentially improve their effectiveness and facilitate their evaluation has been consistently highlighted over the years (Rice & Atkin, 2013; Witte et al., 2001).

### Theoretical Frameworks

Several theoretical models have been found useful in assisting campaign development and evaluation. Two important models are the Extended Parallel Process Model (EPPM) (Witte et al., 2001) and the Elaboration Likelihood Model (Petty & Cacioppo, 1986).

The EPPM focuses on how people perceive health risk information. It is comprised of four main theoretical constructs: susceptibility, severity of a threat, self-efficacy, and efficacy of the recommended response to avert the threat. When people are presented with a message that elicits health risks, they usually start by assessing how susceptible and severe they are to that given health threat. They also evaluate the efficacy of the recommended response to avert the threat and their self-efficacy to carry out the recommended response. Thus, the cognitive outcome of message processing will be a result of simultaneous engagement in efficacy and threat assessment (Witte et al., 2001).

In this sense, if viewers have higher perceived efficacy than perceived threat, they may engage in danger control processes. This type of control process directs the viewer to manage the threat while resorting to the sense of efficacy they hold and are often considered to be adaptive for message processing (Witte et al., 2001). However, if their threat perceptions are higher than their self-efficacy, they may feel overwhelmed and engage in nonadaptive fear control processes. This type of process may lead the viewer to focus on the management of negative emotional states and not resort to his/her perceptions of efficacy to avert the threat. This could lead to unwanted cognitive processes such as denial of the threat, defensive avoidance, and reactance (Witte et al., 2001).

Thus far, only Keller and Honea (2016) have used the EPPM in the field of IPV campaigns targeting females who experience IPV, as part of the formative evaluation research and while pretesting an IPV campaign. The authors conducted interviews and focus groups in which participants were inquired about general perceptions of IPV and the use of support services through questions based on the components of the EPPM. Their findings suggest that it is key to be mindful of gender stereotypes and dynamics in society when developing IPV campaigns targeted at victimized women. Additionally, the systematic content analysis by Reis et al. (2020) suggested that less than 10% of their sample adequately represented the four constructs of the EPPM, as per the recommendations of this theoretical model. To surmount this, their findings indicate that future campaigns could present more information on the susceptibility to the threat and on the efficacy of the recommended response. Nonetheless, to the best of our knowledge, no research has applied its tenets to the actual development and quantitative evaluation of messages directed at men who are the targets of IPV.

The second model that can significantly contribute to campaign development and evaluation is the ELM (Petty & Cacioppo, 1986). The ELM explains how possible attitude change can occur through persuasion. According to ELM, if the recipient of a message is highly motivated and is capable of processing the message, persuasion occurs through what is called the “central route.” In this case, elaboration is relatively high and a thorough analysis of most components in the message is conducted, such as argument strength and complexity, possibly resulting in more stable and long-lasting changes in the recipient. When the recipient is not as capable of processing the message, and/or may also not be as motivated to do so, persuasion may occur through the “peripheral route.” In this low elaboration likelihood route, the receiver employs heuristics to evaluate the message, and for example, he/she may be guided by how credible and similar the communicator of the message is, or the will to solve a dissonant internal state. Thus, different peripheral cues guide their attitudes and beliefs (Petty & Cacioppo, 1986). It is also important to consider that a given persuasion-related outcome often occurs as a result of engagement in both processes, which can shift and combine in complex patterns (O’Keefe, 2013).

Previously, the study by Reis et al. (2020) provided insights about the representation of ELM-related constructs in IPV campaigns directed at men. It found that campaigns often conveyed dissonant states (e.g., describing what the viewer’s situation possibly was, and what it could become if adaptive actions were taken) and portrayed men who could be perceived as normative (i.e., similarity cue). Additionally, the ELM was used specifically for the development of a campaign focusing on domestic violence prevention (Keller & Otjen, 2007). The campaign developed by Keller and Otjen (2007) presented preliminary positive results but focused solely on violence against women.

### Aims of the Current Research

Taking into account the theoretical background on the EPPM and ELM, this study aims to conduct theory-based formative evaluation research to assess overall awareness and impressions of victimized men in different-sex and same-sex relationships regarding existing Portuguese IPV campaigns, as well as their recommendations for the development of new ones.

To achieve this goal, semi-structured interviews were conducted with these populations. Our objective stems from the need to consider the perspective of an intervention’s target population and inquire about their characteristics, predispositions, and impressions about the topic under study, which is in line with formative evaluation research. By considering theory, previous findings from effective health campaign design, and men’s perspectives, we may improve the development and effectiveness of future interventions in this population and potentially reduce unintended negative effects (such as, denial, defensive avoidance, and reactance). Additionally, we produce novel scientific knowledge on this field, by also following the ethical principles on research within vulnerable populations (Roffee & Waling, 2017) and the Journal Article Reporting Standards for the report of primary qualitative research (APA, 2018).

## Method

This study was approved by the Ethical Review Board of its host institution (32/2018). No incentives were provided for participation in this study.

### Sample

Participants were considered if they were over 18 years old and self-identified as males and targets of violence in a past and/or current intimate partner relationship. Both different-sex and same-sex relationships were contemplated, as long as participants spoke Portuguese or English fluently and resided in Portugal. Participants were not included if they were younger than 18 years old and/or reported another type of violence that did not occur in the context of an intimate partner relationship (e.g., minor’s sexual abuse).

Data collection was undertaken before the COVID-19 pandemic, and the call for participants lasted the entire duration of 2019. This call presented the inclusion and exclusion criteria, to which potential participants responded by proactively contacting the researchers demonstrating their interest in participating. They were selected for participation via purposive sampling if they met the inclusion criteria. Data collection ended after preliminary analyses revealed data saturation, which is also in line with findings by Guest et al. (2006) on saturation being achieved with as few as 12 interviews. The call was disseminated through the Portuguese Network for the Support of Domestic Violence Victims with the aid of a governmental organization (CiG). Additionally, it was shared by nongovernmental organizations, such as the APAV, and social networks to reach the most diverse sample possible.

A total of 15 participants were interviewed, but one participant was not included in the analysis because in the later stages of his interview he clarified that he had experienced a form of violence other than IPV. Thus, 14 participants were included for analysis (*M* = 34 years old, *SD* = 8 years old, ranging from 24 to 51 years old). Most participants were single, were working at the moment of participation, and had at least an undergraduate degree. Six participants identified as heterosexual, two as bisexual, and five as gay men. One participant did not disclose his sexual orientation. Only one participant, “O,” was not Portuguese, as he had a migrant background. Our participants were subject to different forms of IPV, namely psychological (e.g., diminishing one’s value), physical (e.g., stabbing), sexual (e.g., forcing sexual contact), and economical (e.g., nonconsensual use of private funds). Relationship duration ranged from 8 months to 19 years, and only one participant remained in the relationship in which he was abused (see Table 1 for an overview of sociodemographic and abusive relationship characteristics).

**Table 1.** Sociodemographic Characteristics.

Variable	*N*	%
Occupational status		
Student	1	7.1
Employed	11	78.6
Unemployed	2	14.3
Education level		
Primary	3	21.4
Secondary	2	14.3
Bachelor’s degree	6	42.9
Master’s degree	2	14.3
Other	1	7.1
Living context		
Urban	11	78.6
Rural	2	14.3
Mixed	1	7.1
Sexual orientation		
Heterosexual	6	42.9
Gay	5	35.7
Bisexual	2	14.3
Not disclosed	1	7.1
Civil status		
Single	11	78.6
Married	1	7.1
Divorced	2	14.3
Has dependents		
No	10	71.4
Yes	4	28.6
Abusive relationship length		
Less than 1 year	3	21.4
Between 1 and 5 years	7	50.0
Between 6 and 10 years	2	14.3
Over 10 years	2	14.3
Filed a criminal complaint		
Yes	8	57.1
No	6	42.9

### Interview Protocol

The interview protocol was semi-structured, and its development was based on the existing literature on IPV, its dynamics and processes, and additional stressors that men in same-sex relationships may experience.^1^ It also considered research on effective health campaign design and the ELM and the EPPM. We opted for the use of a semi-structured approach given the novelty of this topic and to allow for the emergence of other topics that could significantly contribute to our data.

After a final draft was concluded, the protocol was reviewed by three stakeholders: a clinical psychologist, a victim support technician, and an LGBTI activist. The protocol was then pretested with two males and a female who were Psychology researchers and were not part of our sample. These researchers imagined being in a hypothetical IPV situation to further improve clarity and the flow of the script. After this process was concluded, a Portuguese and an English version of the protocol were finalized and were used in all interviews without further changes. The protocol was comprised of both open-ended (e.g., “In your opinion, how is intimate partner violence directed at men seen in today’s society?) and more specific questions (e.g., “How long did this relationship last?). The interview protocol is presented in Supplemental Appendix A.

### Materials

As part of the assessment of impressions on specific Portuguese IPV campaigns, three pictorial campaigns were presented to participants. The posters were printed on an A4 sheet of paper in color and were provided to participants during the corresponding section of the interview. To the best of our knowledge, the authors of this study did not contact the campaigns’ promoting entity, and no information about the campaign development and testing was publicly available.

One of the campaigns, henceforth entitled “X-Ray,” portrayed an X-ray of a person’s skull, highlighting that feeling shame is an invisible yet significant part of being a male and being victimized. Another campaign, “Friends,” presents a “selfie” taken by three people (two men and one woman), in which one of the men has a bruised cheek. Lastly, the campaign “Sweatshirt” portrays a sweatshirt with the word “victim” written on it, stating that anyone can be a “victim” of crime (APAV, n.d.). See Supplemental Appendix D for more details on the materials. These pictorial campaigns were specifically chosen because they presented information that was key to men’s abusive processes, and the information was commonly presented in more generic IPV campaigns, potentially maximizing the external validity of our findings. We highlight that the authors of this study attributed names to each campaign to improve clarity when describing our findings. These names do not represent the actual title or name of the campaigns. Furthermore, to our knowledge, the campaign designated as “X-Ray” is the only IPV campaign in Portugal that has been created exclusively for victimized males. The campaign “Friends” was chosen because it visually depicts bruises on a man while referencing victimization. Finally, the campaign “Sweatshirt” was chosen because we aimed to understand how a more ambiguous message (i.e., not specifically identifying male victimization) was perceived by victimized males.

### Coding Taxonomy Development

The coding taxonomy was both deductively and inductively developed. On a deductive level, we considered the literature on IPV and research on health campaign design, to formulate themes such as the perceived impact and targets of the campaigns. Literature on the EPPM (Witte et al., 2001) and ELM (Petty & Cacioppo, 1986) was also key to inform the creation of specific questions about the four constructs of the EPPM and the characteristics of the person portrayed in the campaign (i.e., a potential heuristic as stated in the ELM).

On an inductive level, the initial coding taxonomy was applied to a small sample of interviews which allowed for the identification of new categories that better represented the data. This inductive level coding was essential to identify constructs that emerged and were not considered in the scopes of the considered theoretical models. By doing so, the plurality of perspectives on these topics was more adequately represented. A final version of the taxonomy was created after its application to all data, as a result of an iterative process of organization and distinction between the categories and their meanings.

### Taxonomy Categories

Our analysis generated 30 categories, 57 subcategories, and 13 (sub) subcategories, applied to 14 units of analysis. These represent a wide range of constructs, such as the needs men had when they were being victimized (i.e., “Men’s needs”) or men’s impressions of the perceived effectiveness of IPV campaigns for the general public (i.e., “Perceived Effectiveness General Public”). A full description of all categories is provided in the Supplemental Appendix B.

Firstly, we addressed the overall awareness about existing campaigns in Portugal, including participants’ recollections of specific characteristics in these campaigns. Our second main group of questions focused on impressions about three specific pictorial Portuguese campaigns. Our categories in this group encompass general evaluation as well as specific impressions about each of these campaigns. We considered the four theoretical constructs of the EPPM and the characteristics of the person portrayed in the message as a possible heuristic from the ELM (i.e., to assess the potential importance of credibility and similarity in persuasion). The last group of questions corresponds to men’s contributions to the development of new campaigns. This group included both general and specific aspects of the campaigns and presented links with previously discussed concepts of the theoretical models under study.

### Coding Process

The coding process followed a post-positivist approach, which highlights the constructivist role that data observation and interpretation have in the sensemaking of a given phenomenon, as well as the importance of anchoring data analysis in theoretical perspectives to achieve a more objective, consensual, and verifiable representation of reality (Panhwar et al., 2017). Thematic analysis was used to code the textual content of the interviews, aiming to identify, analyze, and report patterns within data (Braun & Clarke, 2006). The explicit written content of the interviews was coded, with one researcher coding all interview materials, and the second independent rater coding 50% of these materials. The researcher who conducted the interviews and coded the totality of data has a background in Social Psychology, having obtained specific training in IPV and LGBT-related topics. The other researcher was not familiarized with the topics under study and was provided with the coding taxonomy and its descriptions. Discussions were had to clarify the meanings of certain categories, but we aimed to reduce the potential bias of familiarity with the topic that the second researcher could have. Data were coded using QSR Nvivo 12 software and complementarily organized with Microsoft Excel.

### Risk of Bias Assessment

Interrater reliability was calculated considering different reliability indexes (Cohen’s *K*; Prevalence and Bias Adjusted Kappa [PABAK] and Maximum Kappa—*K*_max_, when applicable). These indexes complement the commonly used Cohen’s *K* and provide information about how interrater reliability may be impacted by variations in prevalence^2^ and bias^3^ between raters (i.e., PABAK), and how the maximum possible K (i.e., *K*_max)_ may be affected by marginal values (Flight & Julious, 2015). In summary, all the previously mentioned measures should be considered when analyzing interrater reliability as they provide different types of information about the same process (Sim & Wright, 2005). For the extrapolation of these indexes, both IBM SPSS (v25) software and the DAG_Stat tool (Mackinnon, 2000) were used. Overall, the mean interrater agreement in our study as stated by Cohen’s *K* was considered to be very strong (Cohen’s *K* = 0.93) according to Landis & Koch’s cutoff proposal (Landis & Koch, 1977). A comprehensive view of these indexes is presented in Supplemental Appendix C showing that overall interrater reliability was very good.

### Procedure

Interviews took place in different locations in Portugal, and in each session in which only the interviewer and the interviewee were present, participants provided their written informed consent after being explained the study’s conditions. All participants consented to the sound recording of their interviews a priori, and the recording equipment was always visible. Firstly, participants were first inquired about their general awareness and recollection of public awareness campaigns that focused on IPV and domestic violence in Portugal. Afterward, they were presented with the three different pictorial campaigns and asked about their impressions. Lastly, participants were questioned about their suggestions for the development of new campaigns. After the end of the interviews, and to avoid potentially negative effects of discussing a sensitive topic, participants were provided with information about help resources. The sessions recorded in audio were transcribed and anonymized for data coding by the independent coder. Only the main researcher had access to and transcribed the sound files that resulted from the interviews. The average duration of the interviews was 2 hours, ranging between 1 hour 30 minutes and 2 hours and 30 minutes.

## Results

The findings of the analysis will be presented in three main sections: knowledge and beliefs about IPV campaigns, impressions about this study’s three poster campaigns, and recommendations for the development of targeted campaigns. Understanding how victimized men viewed and thought of general IPV campaigns before being exposed to the three posters allowed us to make a “baseline” assessment of their beliefs on this topic. It also provided key information about the campaigns’ perceived effectiveness and impact, and what characteristics stood out and were more easily recalled, even if years had passed since the moment of exposure.

### Knowledge and Beliefs About IPV Campaigns and Their Outcomes

This section aims to depict preexisting knowledge and beliefs about the characteristics of Portuguese campaigns on domestic violence/IPV and their potential effects.

#### Lack of awareness about public IPV campaigns directed at victimized men

A small number of participants recalled having seen a campaign on domestic violence or IPV at the time of their victimization, remembering campaigns that focused on female victims of male perpetrators (*n* = 5). For those who remembered being exposed to these campaigns, they considered that the targets were the bystanders since campaigns asked to act when violence is detected by calling a helpline or reporting to the police. Only one participant recalled a campaign specifically directed at victimized men. They recalled seeing these campaigns being shared mostly on television and social media, but billboards, magazines, hospitals, local health clinics, and police stations were also reported.

#### Low perceptions of effectiveness and impact on victimized individuals

Overall, participants felt that the global perceived effectiveness of IPV campaigns was lacking, and different contributing factors were provided. Some participants stated that they did not perceive beneficial outcomes of existing campaigns on their abusive process (*n* = 5), mostly because in their views the campaigns do not lead victimized individuals to act. Additionally, victimized males felt that campaigns did not result in an overall reduction of domestic violence or IPV, but this observation was also attributed to perceptions of ineffectiveness from the legal system to respond to and accommodate those who experience IPV.

#### Campaigns’ potential to break the silence and facilitate help-seeking

Nonetheless, some participants found that campaigns can have some effectiveness in improving different outcomes. For instance, several men mentioned that campaigns could potentially break the silence surrounding male victimization and facilitate help-seeking, namely for those who are at extreme points in their victimization process (e.g., physical violence) (*n* = 10). In this sense, campaigns could work as a starting point, but not as a solution to the problem. Only one participant stated that campaigns let him know there was a way out, informing him that there was someone trained to deal with the issues of IPV.

### Impressions About the Three Pictorial Campaigns

This section aims to depict participants’ impressions about the three pictorial campaigns they were exposed to as part of this study. Only one participant had ever seen one of these campaigns before participating, with the remaining participants not recalling ever being exposed to them previously.

#### Differing perceptions on perceived effectiveness for victimized individuals

Regarding the perceived effectiveness of these specific campaigns for male targets of violence, opinions were evenly mixed. On one hand, some men (*n* = 7) thought that they were effective because they drew attention to male victimization, especially a campaign that specifically stated that men were “victims.” This approach could signal that the organization is aware and prepared to handle male victimization, which along with consistently providing information on helplines might encourage help-seeking. On the other hand, several men (*n* = 5) indicated that they did not strongly believe campaigns would lead targets of violence to act.

#### Clearly directed and explained campaigns that feature “real people.”

Abused men praised messages and slogans that were direct and clear in their purpose and recommended response, in contrast with those which were ambiguous or required more elaboration (*n* = 7). Portraying realistic “everyday people” was rated as best given that it had a higher potential for identification (*n* = 5). Campaigns that looked more modern and featured an overall eye-catching design were evaluated as better. Participants found that campaigns that did not stand out or were poorly executed had reduced appeal (*n* = 4). These findings can be linked to some of the tenets of the ELM and their role in facilitating persuasion. Additionally, some men voiced praise for the depiction of psychological violence, as it remained an under-discussed type of violence (*n* = 3). When faced with a campaign that did not state which type of violence people can experience, one man nevertheless pointed out that this could be beneficial to allow more people to identify with the campaigns.

Not directly stating who was the aggressor was pointed out as a potential drawback by one participant because it often led the viewer toward preestablished notions that did not consider situations such as violence in same-sex relationships. This was further highlighted by the fact that two of the slogans used had considerably different interpretations. Furthermore, one participant specifically expressed his dissatisfaction with the design company that developed the campaign, stating that campaigns should be designed in collaboration with people who had gone through experiences of IPV.

[name of design company] . . . this cannot be done by people who did not go through this, this has to be done by people who went through this because it has to convey a feeling (. . .) this does not convey a feeling. (G, 36 years old)

#### Dissonant views about evoking shame, victimhood, and bruises in campaigns

Several participants commended the portrayal of shame as a mark of the abuse as they felt it adequately represented the experiences victimized men have (*n* = 6).

In the first poster shame as something ‘normal’ in a person, in a man who is a victim of domestic violence helps to understand that it is not needed, or this feeling of shame is not that important to call that number because on the other side there will certainly be a comprehension about all this, something that already helps the person overcome that barrier to be able to call. (E, 35 years old)

In contrast with participants who saw it as a positive aspect, some participants stated that mentioning shame could further stigmatize victimized men, perpetuating stereotypes about masculinity and hindering help-seeking (*n* = 6).

I think it trivializes the matter a lot (. . .) shame isn’t just shame, it is fear, it is depression, it could really be physical marks, so . . . (. . .) it doesn’t correct anything (. . .) if I see this it will not improve my life in any way. (A, 26 years old)

Presenting the word “victim” in the piece of clothing in the “Sweatshirt” campaign also elicited several negative reactions that focused on how the identity of “victim” is something seen as pejorative and that men would not want to be (*n* = 4).

I don’t want to be part of a club (. . .) nobody wants to wear a sweatshirt that says victim so. . . it might fit any person (. . .) but nobody wants to use it, so this doesn’t make sense in my opinion (. . .) it is aggressive. (G, 36 years old)

I think a problem is created with the word victim (. . .) it being associated with a pejorative and an inferior thing, a victim is a person who is below [others] (. . .) a man will not want to be a victim. (B, 23 years old)

Furthermore, the depiction of actual bruises was seen by some as too aggressive or reductive of the entire experience of violence that usually includes psychological violence as well, potentially leading to the invisibility of other types of violence (*n* = 3).

#### EPPM—Lack of information on the effectiveness of recommended responses

Self-efficacy-related information was provided in all campaigns as perceived by most participants, but three participants stated that no self-efficacy information was presented. When asked about whether there was a recommended response to avert the threat present in the campaigns, most participants stated that helplines were suggested (*n* = 9). Nevertheless, a small number of participants considered that no information in the campaigns represented a recommended response to overcome IPV. Subsequently, when asked if there was information available in the campaign about how effective the recommended response was in dealing with the threat, all but one participant indicated that none of the campaigns provided this type of information. The single participant who indicated that there was information on this aspect interpreted the entire campaign as an efficacy source. Another participant voiced concerns that were interpreted as part of this topic when discussing the effectiveness of the campaigns, by questioning what he would gain and what would happen if he called.

What will I gain by contacting [organization]? (. . .) I don’t know what is going to happen, will I stop being a victim? What will I become then? What will I contact you for? (I, 28 years old)

Participants felt that conveying information about statistics could convey threat susceptibility (*n* = 4), but most participants did not recall seeing this information in the campaigns. Threat severity was mostly seen as being present via the bruise in the “Friends” campaign as well as the “X-ray” campaign as it alluded to a clinical situation and suggested psychological suffering (*n* = 6). Conversely, essentially half of the sample did not find that information of victimization severity was present.

### Participant’s Recommendations for the Creation of New Campaigns

When asked about their recommendations for the design of new campaigns targeting victimized men, considering their needs and experience of victimization, men offered different perspectives that will be addressed in the following sub-sections.

#### Providing clear information about violence that is representative of diversity

Explicit information about the different types of violence was considered one of the most relevant types of information across participants (*n* = 7). It could be conveyed in the form of testimonies, reenactments, or simple checklists that would make it easier for victims and bystanders to identify with the situation or have concrete information available. Due to the complex nature of victimization, specific information should address how hard it is to identify violence as violence, and what are its early signs. Mainly gay and bisexual participants emphasized the importance of portraying nonheterosexual realities, to increase public awareness about violence in same-sex relationships (*n* = 4).

(. . .) the most important is (. . .) for the campaigns to have an instructive impact and educate people that these cases exist (. . .) be it [violence] from a man to a woman, a woman to a man, a man to another man, a woman to another woman. (E, 35 years old)

Information regarding legal rights was not found very adequate by some participants (*n* = 4). In what concerns the type of channels to be used, video formats were emphasized as potentially more appealing than pictorial formats by three participants.

#### Portraying normative men

As postulated in the ELM about the potential role of message source similarity and credibility as a persuasion heuristic, most participants would portray males similar to them on the campaigns, providing testimonies, for example, to not only improve the credibility of the campaign but also to improve identification (*n* = 8).

(. . .) testimonies, people who went through this and lost their shame, to help others” (L, 42 years old)

(. . .) there had to be a resemblance, (. . .) a person could look and identify with that character, that man, and have some empathy and think, I look like him. (G, 36 years old)

Other suggestions were using physicians who advised or recommended something (*n* = 4), and lastly, two participants mentioned the possible use of celebrities. The people portrayed should represent the LGBT community as well, as stated by gay and bisexual participants (*n* = 4).

##### Appealing to emotions

Some participants preferred a more emotional message when compared with a more logical or rational one (*n* = 5). This would mean that campaigns would have to capture participants’ attention by triggering pertinent emotions in their processes. Three participants preferred a mixed approach with both types.

#### EPPM—Feeling susceptible, empowered, and understanding what happens when help is sought is key

Participants’ suggestions about the content of future campaigns were also in line with the four theoretical constructs of the EPPM, which will be presented in the following subsections.

Threat susceptibility was seen by most men as an important factor of the EPPM to be present in new campaigns (*n* = 8). Men suggested that statistics on the prevalence of violence directed at men could inform not only men that they are not the only victimized ones but also inform bystanders. Self-efficacy was highlighted as an important resource and oftentimes crucial in determining help-seeking efforts (*n* = 8). Other participants mentioned that information regarding self-efficacy should be more noticeable in comparison with other elements of the campaign (*n* = 3).

(. . .) give power to the person to at least know where to call to help deal with the situation. (M, 38 years old)

(. . .) the empowerment to call a helpline or the empowerment to leave these situations would be really important because that’s the turning point for any person that may be going through a situation like this. (E, 35 years old)

Several participants reported that it was important to provide information on helplines (*n* = 8). Efficacy of recommended response was stated as an important component of future campaigns, explaining the psychological benefits of calling, what happens when people seek help, and in what circumstances it applies. One participant felt that recommended responses were not that important, and another mentioned that conflating information on violence and helplines might hinder its capacity.

Several participants indicated that presenting information on the serious physical, psychological, social, and financial consequences of violence was important (*n* = 6). Social isolation was highlighted as a particularly important consequence given that it was felt by several participants and impacts the sense of invisibility of the violence experienced, which leads to more invisibility of men who are victimized. Consequences on future relationships, dependents, and job performance were also mentioned.

## Discussion

This qualitative study aimed to conduct formative evaluation research with victimized men in different-sex and same-sex relationships to understand their overall knowledge and impressions about existing Portuguese pictorial IPV campaigns. Additionally, it collected their needs and recommendations for the development of new campaigns targeting victimized males. Our analysis, which took into consideration the literature on IPV, the ELM, and EPPM, provides important results that are the first of its kind and are a significant contribution to the field of male victimization and public awareness campaigns for this population.

In what concerns overall awareness and beliefs regarding IPV campaigns in Portugal, only one of our participants recalled ever seeing a campaign directed at victimized men. Additionally, men voiced that they did not see campaigns as effective in leading to actual behavior change nor did they feel that these campaigns helped them in their abusive process. This may be due to the lack of visibility surrounding male victimization in different-sex and same-sex relationships (Edwards et al., 2015; Laskey et al., 2019), something expressed in this study as an issue campaigns could intervene on. Furthermore, there is a documented lack of awareness-raising efforts toward victimized males (Hine et al., 2020; Martín et al., 2019), which may be reflected in Portugal due to the small number of campaigns focusing on victimized men (APAV, n.d.; Commission for Citizenship and Gender Equality, n.d.-b). Thus, future campaigns could be directed at victimized men in different-sex and same-sex relationships, informing targets of violence as well as increasing awareness in society about this issue. By doing so, crucial information on topics such as what help resources are available, as well as how effective they are in helping those victimized could be provided. This could lead to the correction of misconceptions (e.g., perceptions of the ineffectiveness of the legal system in handling IPV cases), which in turn could potentially lead to better attitudes, perceived effectiveness, and actual help-seeking behaviors. We must nevertheless highlight that to our knowledge a unified and consensual definition of “perceived effectiveness” in the covered literature remains to be developed. This is due to discrepancies in the theoretical underpinnings, study designs, and methodologies used in this field, which limit the comparison of results across studies (Noar, 2006).

Regarding the impressions on specific campaigns, on one hand, it should be highlighted that many positive aspects were found, and that if adequate, could be replicated in future campaigns. Overall men seem to prefer messages that are clear and direct, and that identify the different possible types of violence. Importantly, most participants perceived that self-efficacy was present in all campaigns. This construct is central in leading to attitude and behavior change by theoretical frameworks such as the EPPM (Witte et al., 2001) and the Theory of Planned Behavior (Ajzen, 1991) and has been previously suggested to be central in campaigns directed at victimized women (West, 2013). On the other hand, some topics are deserving of more attention by future intervention developers, namely when covering topics such as shame, using the word “victim” and depicting explicit bruises or wounds.

Regarding shame, the literature has highlighted it to be a common experience in victimized females (McCleary-Sills et al., 2016) and males (Hine et al., 2020). Developers should be cautious not to further stigmatize those who have and could be currently struggling with this issue, as previous research with victimized women suggests (West, 2013). The use of the word “victim” should also be thought of carefully, as previous literature on IPV reveals the struggles of those who experience stigma and in coming to terms with their identities (Eckstein, 2010). The intersection of stigmatized identities, hegemonic masculinity norms, and LGBT-specific processes (Stiles-Shields & Carroll, 2015) plays an important role in men’s abusive and help-seeking processes (Eckstein, 2010) and should be considered when designing interventions.

Presenting explicit bruises and wounds also received mixed responses, and this may echo the findings by West (2013) with female targets of violence, and previous analyses of campaigns directed at the same population (Magaraggia & Cherubini, 2017). If for once it could be used as an attention-grabbing visual cue according to the ELM (Petty & Cacioppo, 1986), without further testing, researchers should be mindful of how these graphic depictions might be more harmful than beneficial, leading people to deny or avoid the message (Witte et al., 2001). Nevertheless, researchers and designers can investigate what other types of information could be efficient to elicit threat severity in the target audience without the need for actual depictions of violence.

Formative evaluation research should also be conducted to pretest the slogans and information presented, as in our study men did not entirely agree on a single interpretation, leaving room to improve their clarity and degree of understanding (Lee & Kotler, 2019). This can also potentially be a heuristic cue, as stated in the ELM, given that complex arguments may require higher cognitive elaboration, and as a consequence may discourage viewers to process and integrate the message (Petty & Cacioppo, 1986). All of this may have contributed to the mixed perceived effectiveness of these campaigns for male targets of violence, which is in line with previous qualitative (West, 2013) and quantitative findings (Campbell & Manganello, 2013).

Most participants did not perceive several constructs of the EPPM to be present, such as the effectiveness of the recommended response (i.e., helplines), which is in line with Reis et al.’s (2020) conclusions and could be crucial in improving awareness about help services and what they can provide. The fact that men felt that campaigns do not lead people who experience IPV to act may also be explained by many factors that are independent of campaigns. Leading people to act based on messages is a multifaceted issue that has been the focus of decades of research in health promotion (Abroms & Maibach, 2008), and the fact that people are in a situation of added vulnerability and potential danger adds a layer of complexity to this topic.

Nevertheless, on a more subjective level, but that is inherently linked with the principles of formative evaluation research, we highlight that one man voiced that efforts such as campaigns should be developed “by people who went through” violence. Not only does this potentially add to the effectiveness of the campaigns, potentially reducing unwanted negative effects, but it is an important ethical consideration when researching populations who have been consistently victimized and made invisible (Roffee & Waling, 2017).

Finally, our sample of men who were the targets of IPV offered their insights regarding the development of new campaigns directed at victimized men. Men would design these campaigns to feature information on different types of violence and how to identify its early signs. One crucial aspect that could potentially serve as a cognitive heuristic according to the ELM (Petty & Cacioppo, 1986) is their suggestion for the portrayal of men “like them,” someone whom they could see themselves in. The role of message source similarity and credibility in potentially increasing persuasion has been documented previously and could be explored in future research (Jones et al., 2003; Rice & Atkin, 2013). Previous research of pictorial campaigns directed at victimized men highlighted that mostly normative, young, and White males were represented (Reis et al., 2020), but this is a very limited representation of males who experienced IPV. Thus, we highlight the need for adequate and meaningful representation that encompasses men who are in same-sex relationships, men of different ages, ethnicities, degrees of functionality, as well as other individual or cultural characteristics. Considering different languages other than the countries’ native language is also important as one man did not speak Portuguese and did not understand the campaigns’ content until it was translated to him.

Regarding the constructs of the EPPM, victimized men found essentially all to be important to be presented in future campaigns, with an emphasis on threat susceptibility, which is in line with recommendations by Reis et al. (2020). Nevertheless, pushing for these constructs should be done while pretesting these stimuli in order not to generate unwanted negative responses.

### Limitations

This study has different limitations that could be improved upon in future research. Firstly, in our data collection, we could not gather the impressions of older victimized males and/or trans men. This may be due to the added invisibility and stigma of IPV in these populations, but gathering their impressions is central for understanding their specific circumstances of violence and the development of specific interventions for these populations. Secondly, participants’ victimization frequency and severity ranged considerably, which could influence their impressions. Future studies could try to discern how different degrees of victimization frequency and severity influence perceptions of campaigns. In line with this, participants were asked to recall memories in negative circumstances of their lives, which may have impacted the accuracy of recall. Thus, a third limitation pertains to our retrospective methodological approach, which could be improved by including the specific assessment of how memory may be impacted by IPV. Fourthly, our study allowed participants to view all aspects of each campaign without a time limit, which may not be representative of actual real-life contexts. Future studies could study recollection of campaigns in real-life contexts after implementation and select different types of campaigns. Additionally, other theoretical models such as the Health Belief Model (Rosenstock, 1974) could be pertinent for the study of the topics at hand. Lastly, we would like to highlight that the authors did not contact the campaigns’ promoting entity and were not aware of the different considerations taken during campaign development, or if any evaluation research was carried out. It is possible that some aspects of the campaigns design, mentioned in this article, were covered during the development of the campaigns under study.

## Conclusion

IPV negatively impacts the lives of women and men worldwide, but research thus far has scarcely addressed the victimization of males and the development of targeted interventions for them. This study presented formative evaluation research conducted with victimized males in abusive different-sex and same-sex relationships to collect information about their awareness, beliefs about IPV campaigns directed at men who were targets of IPV, and recommendations for the creation of new campaigns. To the best of our knowledge, this is the first research to investigate awareness campaigns targeted at abused men taking into account their feedback.

Overall participants did not recollect IPV campaigns, nor did they feel these campaigns had any influence on their abusive process. In terms of campaign content, men seem to prefer direct and concise information related to different types of violence, the portrayal of realistic men such as them, and the presence of information regarding threat susceptibility, self-efficacy, and efficacy of the recommended response in the campaign. Our findings seem to corroborate some results obtained with abused women but also add a significant amount of theoretically grounded considerations that campaign developers should consider in future efforts.

It is not possible to create universally accepted campaigns, but our findings suggest the multiplicity of perspectives the same pieces of information may have. Our results highlight the need to continue using theory-based formative evaluation research and pretest of materials before dissemination in a population that is already victimized and in a situation of added vulnerability. Future research could aim to improve on the lack of research on this topic and population and to investigate if and how to frame more sensitive topics such as shame and victimhood while exploring how to address samples of diverse men.

## Supplemental Material

sj-docx-1-jiv-10.1177_08862605221108075 – Supplemental material for Men Who Suffered Intimate Partner Violence: Impressions About Existing Public Campaigns and Recommendations for New Ones Click here for additional data file. Supplemental material, sj-docx-1-jiv-10.1177_08862605221108075 for Men Who Suffered Intimate Partner Violence: Impressions About Existing Public Campaigns and Recommendations for New Ones by Eduardo Reis, Patrícia Arriaga and Carla Moleiro in Journal of Interpersonal Violence

## Notes

1. Data collected pertaining to these topics will be the focus in future research.

2. Prevalence in this context pertains to the probability with which a rater classifies a portion of data as “one,” in contrast with rating it as “zero.”

3. Bias relates to the symmetry of the rating table and is linked with the frequency at which raters choose a particular classification over others (i.e., a non-biased table will be symmetrical) (Flight & Julious, 2015).
