# Supplementary material for: Men Who Suffered Intimate Partner Violence: Impressions About Existing Public Campaigns and Recommendations for New Ones
Source: J Interpers Violence. 2022 Aug 3;38(3-4):3534–62. doi: 10.1177/08862605221108075 (PMC9850386; doi:10.1177/08862605221108075)
Supplement: sj-docx-1-jiv-10.1177_08862605221108075 – Supplemental material for Men Who Suffered Intimate Partner Violence: Impressions About Existing Public Campaigns and Recommendations for New Ones [file sj-docx-1-jiv-10.1177_08862605221108075.docx]

**Appendix A**

*Interview protocol*

| Main Questions | Follow-up questions |
| --- | --- |
| During your circumstances of violence had you ever seen / heard of IPV campaigns? | What do you remember? |
|  | Do you remember any campaigns for men who are targets of violence? |
|  | Who was the person portrayed as the target of violence? |
|  | Through what channels did you have access to these campaigns? |
|  | Did these campaigns provide a way to solve the problem presented in the campaign? |
|  | Have these campaigns played any role in your situation of violence? |
| Do you feel that these campaigns result in different outcomes? | Do they translate into something objective in the targets of violence? |
| What do you think of each of these images? | What are the positive aspects for a man who is being targeted with violence? |
|  | What are the negative aspects for a man who is being targeted with violence? |
|  | What are the positive and negative aspects for the general public? |
|  |  |
| Do any of these campaigns recommend a recommended response to help you resolve the situation described in the campaign? |  |
| Do any of these campaigns give you information about how effective this recommended response is in solving the problem? |  |
| Do any of these campaigns enhance the feeling that you are capable of performing this recommended response? |  |
| Do any of these campaigns inform you of the susceptibility to the problem portrayed? |  |
| Do any of these campaigns inform you of the severity of the presented problem? |  |
| Do you consider them effective in giving men who are targets of violence important information to assist them in the process of violence? | And for the general public? |
| What attributes would the new campaign that you would create have? | What kind of information should it have? |
|  | Should people be portrayed? If so, who? |
|  | More emotional, or rational? |
|  | Regarding the previously discussed topics on efficacy and risk, are there any that you think are important for men who are being targets of violence? Could you provide examples of content for these topics? |

**Appendix B**

*Coding Taxonomy*

| Theme | Category | Subcategory | (sub) Subcategory | Definition |
| --- | --- | --- | --- | --- |
|  | Identities |  |  | Encompasses men's insights into what being a man means, how it intersects with being a "victim" or not, among other topics related to identities in this specific part of the interview. E.g. "*Victim just on its own I think it doesn't work well, because here we say, this is a mark of a man who is a victim of domestic violence, and says, who is, is the man, what was the victim, domestic violence, only victim, victim is a weak person, the idea is still that idea that the victim is weak* (…)" |
| Knowledge of Previous Campaigns | Beliefs about Masculinity Identities |  |  | References to general beliefs about what it means to be masculine in society, what sort of expectations are associated with it, and what consequences it might have in people's lives that were provided as a reply to the lack of awareness of interventions targeted at victimized men in Portugal. Masculinity was linked with a lack of visibility on IPV directed at men, as well as specificities when developing future campaigns and interventions. E.g. " (...) *we should let go of prejudice, or manage to take it away, or facilitate things a bit more, in a way that men can denounce or have the courage to, I suffer this and this* (…)". |
|  | Beliefs about Society and Victimization |  |  | References to general beliefs about what abuse entails and identities such as victim and survivor. Perceptions about the current state of society in what concerns gender, violence, media and more. These references were observed when men provided their explanations for the lack of public investment and campaigns directed at men, and what could be done to facilitate it. E.g. "*yes, after it was, but I always saw related to domestic violence against women, I never saw in relation to men, because as well, just like I said to [organization], the news are only focused on women, but I have never seen a news piece about a man who was killed by his wife* (…)" |
|  | Characterization of Previous Campaigns | Channels |  | References to which channels were used to convey the campaigns participants recalled. E.g. "(…) *television, outdoors on the street*"; "*I only saw not so long ago, some posters on Facebook, I think on Instagram too*." |
|  |  | Perceived Targets |  | References to who were the perceived targets of the campaigns participants recalled. Could be women, men, or others. E.g. "(…) *for men no, I don't really remember anything*" |
|  |  | Target of Violence Portrayed | Children | This code encompasses references to children as the target of violence portrayed in the campaigns participants recalled. |
|  |  |  | Men | References to men as the target of violence portrayed in the campaigns participants recalled. |
|  |  |  | Women | References to women as the target of violence portrayed in the campaigns participants recalled. E.g. "(…) *women being battered and that they can denounce*"; " (…) *the man and the woman* (…) *yeah, different posters, two posters if I'm not mistaken*.". |
|  |  |  | Other | References to other people as the target of violence portrayed in the campaigns participants recalled. E.g. "(…) *campaigns also for children and elders.*". |
|  |  | Recollection and characteristics |  | References to recollected characteristics of the campaigns participants mention. E.g: " *No, at the time it wasn't like this, booming like it is now. Even if it were perhaps I wouldn't pay much attention*". |
|  |  | Evaluation |  | References to positive or negative aspects of the campaigns participants recalled. E.g. "*I think that the fact that it is starting to get talked about I think it is very little...*"; " (…) *yes, facilitates. [imperceptible] to deal with the matter*." |
|  |  | Recommended Response |  | References to the recommended response provided in the campaigns participants recalled. E.g: " (…) *it would be more the existence of a helpline, if I need anything the number is there*"; |
|  |  | Suggestions for Improvement |  | This code encompasses references to suggestions for improvement of future or existing campaigns. |
|  | Perceived Campaign Effects | Outcomes on General population and targets of DV and IPV |  | References about the general impressions on the effectiveness (or lack thereof) of DV or IPV campaigns on female and male targets of violence. E.g. "(…) *at least bring it to your attention, yes* (…)" |
|  |  | Outcomes on Interviewee’s Abusive Process |  | References to how campaigns impacted the participants' own abusive process. Specific outcomes such as information about helplines can be considered. E.g. " (…) *know what I can do, know that I have a choice there, the alternative, that someone specialized exists to help*" |
|  |  | Perceived Effectiveness |  | References to the general perceived effectiveness of the campaigns participants recalled. E.g. "(…) *men and women, I don't think so*." |
| Impressions on existing campaigns | Knowledge |  |  | References to general knowledge on campaigns, such as recalling their existence and other aspects related to public prevention efforts. E.g. "(…) *no, because it is [organisation], I trust there are good people there*." |
|  | Aspects for the General Public | Negative |  | References to negative aspects that the campaigns may have for the general public. E.g. "(…) *indeed, it makes it seems, I think it reinforces the view that a man is strong and can defend himself if he wants to, and if he doesn't defend himself feels ashamed because of it*." |
|  |  | Positive |  | References to positive aspects that the campaigns may have for the general public. E.g "(…) *having awareness that this happens, for example this one with the sweatshirt that can fit any person, I say that it is generic, that it is a man or a woman*." |
|  |  | Other |  | References to other aspects that the campaigns may have for the general public, that cannot be listed as entirely positive or negative. E.g. “*Ok, maybe this one will go like, the one with the shame, x-ray, going towards that idea that men do not denounce because they are ashamed to do so*" |
|  | Aspects for Targets of Violence | Negative |  | References to negative aspects that the campaigns may have for the targets of violence. E.g. "*The first one, it makes it seem like there is no physical damage, the x-ray one"; "I don't see negative aspects*". |
|  |  | Positive |  | References to positive aspects that the campaigns may have for the targets of violence. E.g. "*Having someone that is also going through this, which is destigmatizing, that I am not the only one, there are more men*". |
|  |  | Other |  | This code encompasses references to other aspects that the campaigns may have for the targets of violence, that cannot be listed as positive or negative. E.g. " (…) *that for the person him/herself, to others, having indicators, have a sort of checklist where there are symptoms, what the person displays...*" |
|  | Campaigns | “Friends” | Negative | References to negative aspects that the campaign that portrays three people. These references might be the result of a direct question, or general impressions provided by the participant that are considered to be negative. |
|  |  |  | Positive | References to positive aspects that the campaign that portrays friends. These references might be the result of a direct question or general impressions provided by the participant that are considered to be positive. |
|  |  |  | Other | References to other aspects of the campaign that portrays three friends. These references might be general impressions provided by the participant that aren't considered to be negative or positive. E.g. "*This is a bit of marketing, that is, it is a marketing perception, I understand that here you have to send the message across to both sides, I'm not even saying...*" |
|  |  | “Sweatshirt” | Negative | References to negative aspects that the campaign that portrays a sweatshirt. These references might be the result of a direct question, or general impressions provided by the participant that are considered to be negative. |
|  |  |  | Positive | References to positive aspects that the campaign that portrays an x-ray. These references might be the result of a direct question or general impressions provided by the participant that are considered to be positive. |
|  |  |  | Other | References to other aspects of the campaign that portrays a sweatshirt. These references might be general impressions provided by the participant that aren't considered to be negative or positive. E.g. "*The third one, the third one with the shirt of the victim, encompasses all kinds, all kinds of violence in this case, all genders, of the people who can be victims*". |
|  |  | “X-Ray” | Negative | References to negative aspects that the campaign that portrays an x-ray. These references might be the result of a direct question, or general impressions provided by the participant that are considered to be negative. |
|  |  |  | Positive | References to positive aspects that the campaign that portrays an x-ray. These references might be the result of a direct question or general impressions provided by the participant that are considered to be positive. |
|  |  |  | Other | References to other aspects of the campaign that portrays a x-ray. These references might be general impressions provided by the participant that aren't considered to be negative or positive. |
|  | Extended Parallel Process Model | Threat Susceptibility |  | Definition: An individual's perception of the likelihood of being adversely affected by the occurrence of a threat. E.g."*I think so, I think so. Statistics and all that, they don't mean anything. People are told their mortgage is 60% and they don't care*" |
|  |  | Threat Severity |  | Definition: An individual's perception of the seriousness or significance of a threat. E.g. "*Yes, that it is not an uncommon situation, that it has consequences, and it would be more in the sense of, ok always having the number right, but of creating more awareness for the people surrounding the victims…*"; "*I think at that time probably, the last one you said, you can do it, but now looking back I think it was more important to understand the consequences that this brings*" |
|  |  | Self-Efficacy |  | Definition: The individual's perceived ability to perform the recommended response. E.g. "*I think so, I think because people in these phases feel that they have none of that, but at the same time it can also repel, I'm thinking, ok, I feel like crap, I feel like I can't do it nothing and they are telling me, and it depends on the message, it depends on the message that will be there*" |
|  |  | Recommended Response |  | Definition: specific responses that could be recommended to overcome the threat. E.g. "*Yes, it is not an unusual situation, it has consequences, and it would be more in the sense of ok always the number right, but more of creating awareness for the people surrounding the victims ...*"; “*What will I gain by contacting [organization]?* (…) *I don’t know what is going to happen, will I stop being a victim? What will I become then? What will I contact you for?*” |
|  |  | Efficacy of Recommended Response |  | Definition: The perceived effectiveness of the recommended response in averting the threat. E.g. "*Yes, and maybe i can call because i have a friend who is probably doing that, what should i do…*"; "*I don't care that 90% of the cases we deal with have been successful, I don't care about that, I'm interested in my case, I in particular, who is yours to contact, what will I have?*" |
|  | LGBT |  |  | References about any LGBT related topics or subjects for this part of the interview. E.g. "*No no. I think that if a man is victimized by another man he will understand the message here*."; "*Yes I think that they should, they should make campaigns about that, people who are homosexuals, LGBT they should do that, to allow so that other people can open a bit of...*" |
|  | Other |  |  | References that could not be attributed to other existing codes for this portion of the interview. E.g “*[name of design company]… this cannot be done by people who did not go through this, this has to be done by people who went through this because it has to convey a feeling (…) this does not convey a feeling*.” |
|  | Perceived Effectiveness | General Public |  | References to the campaign's perceived effectiveness for the general public. May include information regarding their outcomes on this population. E.g. "*Yes, this one has anyone can be a victim of crime and its seems to me more directed at empowering, but still..."* |
|  |  | Targets of Violence |  | References to the campaign's perceived effectiveness for the targets of violence. May include information regarding their outcomes on this population. |
|  | Person | Celebrity |  | References about the use of celebrities as persuasive agents in campaigns, whether in favour or against. E.g. "*Perhaps a celebrity that supports the cause..*.". |
|  |  | Medic |  | References about the use of medics as persuasive agents in campaigns, whether in favour or against. E.g. "*Someone that works at [organisation], a psychologist, a physician*". |
|  |  | Regular Person |  | References about the use of regular persons as persuasive agents in campaigns, whether in favour or against. E.g. "*So it could really be me here, or a friend I: ok so what kind of people should be present in these campaigns in your opinion?*; “(…) *people that I find in my daily routine*." |
|  |  | Other |  | References about the use of entities other than celebrities, medics or regular persons as persuasive agents in campaigns, whether in favour or against. E.g. "*Yes, or someone from [organisation], or someone from the police, or someone that effectively deals with this*". |
|  | Type of Appeal | Emotional |  | References to the impressions on emotional content present or not in the campaigns. E.g. "*Yes, maybe on the side of, go more for the emotional side, I don't know if with the person that is going through this and I'm once again trying to go back to then once again..."* |
|  | Type of Information | Isolation |  | References to isolation and how it may affect men who are targets of violence, and how it could play a role in campaigns. E.g. "*Yes, because that is psychological violence...*" |
|  |  | Nature and Types of Violence |  | References to the nature and types of violence how they could play a role in campaigns. E.g. "*Show that everyone is a victim, or that anyone could be a victim, because I think what I couldn't understand at that time, was that that was violence, that was me being a victim*"; “(…) *one day I had black eyes and I remember that I took a photo because some friends of mine were taking a photo for [social media] but we didn’t even publish it because I asked them not to publish it* (…) *I took the photo, I have black eyes and I am laughing, you get it*?”. |
|  |  | Social Processes |  | References to social support, social influence and its processes, such as stigma, discrimination, considering the subjective norm, and how it could play a role in campaigns. E.g. "(…) *In that, the social support of the person, it is highlighted that this happens, this issue occurs... So that people can, so that it can open their eyes, have the consciousness of what is going on to be able to look at things and identify them*." |
|  | Visual Components | Captivating |  | References to how and which campaigns were most captivating for the participants. E.g. "*The one with the bruises, of the ‘tags’, yes*." |
|  |  | Color Variation |  | References to colour variation and its possible contribute to campaign design. E.g. "*The x-ray maybe draws more attention* (…) *it is something that we associate with cases of extreme violence, going to the hospital*". |
|  |  | Image Composition |  | References to how the different campaigns framed the information in text and images, and other aspects such as ideas about possible articulations of these concepts. E.g. "(…) *it is a funny image, because of the way it is constructed, now I don't believe that it has an effect*..." |
|  |  | Type of text |  | References to text size and its variation in the campaigns. E.g. "*It's on a correct level. Yes*". |
| Creation of New Campaigns | Beliefs about Masculinity and Victimization |  |  | References to men's insights into what being a man means, how it intersects with being a "victim" or not, among other topics related to identities. E.g. "*Victim just on its own I don't think it works well, because here we say , this is the mark of a man victim of domestic violence, and it says, who is, the man, was victim of what, domestic violence, just victim, victim is a weak person, that’s the idea that we still hold that the victim is weak..."; “I don’t want to be part of a club (…) nobody wants to wear a sweatshirt that says victim so… it might fit any person (…) but nobody wants to use it so this doesn’t make sense in my opinion (…) it is aggressive*”; “*I think a problem is created with the word victim (…) it being associated with a pejorative and an inferior thing, a victim is a person who is below [others] (…) a man will not want to be a victim*”. |
|  | Extended Parallel Process Model | Threat Susceptibility |  | Definition: An individual's perception of the likelihood of being adversely affected by the occurrence of a threat. E.g."*I think so, I think so. Statistics and all that, they don't mean anything. People are told their mortgage is 60% and they don't care*" |
|  |  | Threat Severity |  | Definition: An individual's perception of the seriousness or significance of a threat. E.g. "*Yes, that it is not an uncommon situation, that it has consequences, and it would be more in the sense of, ok always having the number right, but of creating more awareness for the people surrounding the victims…";* "*I think at that time probably, the last one you said, you can do it, but now looking back I think it was more important to understand the consequences that this brings*" |
|  |  | Self-Efficacy |  | Definition: The individual's perceived ability to perform the recommended response. E.g. “(…) *give power to the person to at least know where to call to help deal with the situation*.”; “(…) *the empowerment to call a helpline or the empowerment to leave these situations would be really important because that’s the turning point for any person that may be going through a situation like this*“ |
|  |  | Recommended Response |  | Definition: specific responses that could be recommended to overcome the threat. E.g. "*Yes, it is not an unusual situation, it has consequences, and it would be more in the sense of ok always the number right, but more of creating awareness for the people surrounding the victims ..*."; "*I think it should appear in a second moment, not appear all at once, that is, for example, there are these three or four types of violence, it appears right after contacting ..*." |
|  |  | Efficacy of Recommended Response |  | Definition: The perceived effectiveness of the recommended response in averting the threat. E.g. "*Yes, and maybe i can call because i have a friend who is probably doing that, what should i do…";* "*I don't care that 90% of the cases we deal with have been successful, I don't care about that, I'm interested in my case, I in particular, who is yours to contact, what will I have?*" |
|  | LGBT |  |  | References about any LGBT related topics or subjects for this part of the interview. E.g. "*Here when I'm imagining people but with, that you could notice bodies and not necessarily Manuel or Luís...*"; “(…) *the most important is* (…) *for the campaigns to have an instructive impact and educate people that these cases exist* (…) *be it [violence] from a man to a woman, a woman to a man, a man to another man, a woman to another woman*” |
|  | Other |  |  | References that could not be attributed to other existing codes for this portion of the interview. E.g "*I continue to have my opinion that I will repeat again, I think we in this, should have a specialized department, be it in the police force...*"; “[*name of design company]… this cannot be done by people who did not go through this, this has to be done by people who went through this because it has to convey a feeling* (…) *this does not convey a feeling.*” |
|  | Person | Celebrity |  | References about the use of celebrities as persuasive agents in campaigns, whether in favour or against. E.g. "*Perhaps a celebrity that supports the cause...*". |
|  |  | Medic |  | References about the use of medics as persuasive agents in campaigns, whether in favour or against. E.g. "*Someone that works at [organisation], a psychologist, a physician*". |
|  |  | Regular Person |  | References about the use of regular persons as persuasive agents in campaigns, whether in favour or against. E.g. "*So it could really be me here, or a friend I: ok so what kind of people should be present in these campaigns in your opinion? P: people that I find in my daily routine*."; “(…) *testimonies, people who went through this and lost their shame, to help others*”; “(…) *it had to be an identification,* (…) *a person could look and identify with that character, that man, and have some empathy and think, I look like him*” |
|  |  | Other |  | References about the use of entities other than celebrities, medics or regular persons as persuasive agents in campaigns, whether in favour or against. E.g. "*Yes, or someone from APAV, or someone from the police, or someone that effectively deals with this*". |
|  | Type of Appeal | Emotional |  | References to the impressions on emotional content present or not in the campaigns. E.g. "*Yes, maybe on the side of, go more for the emotional side, I don't know if with the person that is going through this and I'm once again trying to go back to then once again...*" |
|  |  | Rational |  | References to the impressions on rational content present or not in the campaigns. E.g. "*Well, I like numbers right, so...*"; "*It is not something completely deprived of emotionality, but yes, informative and less emotional sure*". |
|  |  | Other |  | References to including other kinds of content in campaigns. |
|  | Type of Information | Isolation |  | References to isolation and how it may affect men who are targets of violence, and how it could play a role in campaigns. E.g. "*Yes, because that is psychological violence...*" |
|  |  | Legal Components |  | References to the legal components involved in domestic violence/intimate partner contexts, and how it could play a role in campaigns. E.g. "S*o the person talking about going to courts and such things, I think, I think that’s a step backwards*." |
|  |  | Social Support, Influence and Processes |  | References to social support, social influence and its processes, such as stigma, discrimination, considering the subjective norm, and how it could play a role in campaigns. E.g. "(…) *in that, the social support of the person, it is highlighted that this happens, this issue occurs... So that people can, so that it can open their eyes, have the consciousness of what is going on to be able to look at things and identify them*." |
|  |  | Types and Nature of Violence |  | References to the nature and types of violence how they could play a role in campaigns. E.g. "*Show that everyone is a victim, or that anyone could be a victim, because I think what I couldn't understand at that time, was that that was violence, that was me being a victim*". |
|  | Visual Components | Color Variation |  | References to colour variation and its possible contribute to campaign design. E.g. "*The x-ray maybe draws more attention I: the black - P: it is something that we associate with cases of extreme violence, going to the hospital*". |
|  |  | Image Composition |  | References to how the different campaigns framed the information in text and images, and other aspects such as ideas about possible articulations of these concepts. E.g. "*... it is a funny image, because of the way it is constructed, now I don't believe that it has an effect...*" |
|  |  | Type of Text |  | References to text size and its variation in the campaigns. E.g. "*It's on a correct level. Yes*". |
| Helplines | Beliefs |  |  | References to general beliefs about helplines, their outcomes and effectiveness in aiding those who use them. E.g. "(…) *the first action will go this way, and if on the other side there is someone that empowers us to denounce, someone that properly explains, of how we should do things, maybe we will do*". |
| Abuse | Men’s needs |  |  | References to the different needs expressed by men in their situations of abuse, or what would have helped them in retrospective. E.g. "*To me what was really necessary was talking with someone and that person at the that time recognize that the problem was valid*". |
|  | Shame |  |  | References to feelings of shame by the targets of violence, or other considerations regarding how shame played a role in their process. E.g. "*I don't know, well, I was never ashamed of what happened right, but it was something I hid because I wanted to, so...*"; “*In the first poster shame as something ‘normal’ in a person, in a man who is a victim of domestic violence helps to understand that it is not needed, or this feeling of shame is not that important to call that number because on the other side there will certainly be a comprehension about all this, something that already helps the person overcome that barrier to be able to call*” |
|  | Victimhood |  |  | References to the nature and processes associated with victimhood, considering or not its adequacy in explaining the participants' experiences as they were abused. E.g. "*I don't feel like a victim just because I allowed it to happen to myself*". |
|  | Other |  |  | References that do not fit the other categories. E.g. “(…) *I was provoked for physical violence by psychological violence.*" |

**Appendix C**

*Detailed overview of interrater reliability indexes for each code*

| Code Definition | Cohen’s Kappa | Observed Agreement | Chance Agreement | Positive Agreement | Negative Agreement | Prevalence & Bias Adjusted Kappa | Byrt’s Prevalence Index (PI) | Byrt's Bias Index (BI) |
| --- | --- | --- | --- | --- | --- | --- | --- | --- |
| Abuse – Men’s Needs | 1 | 1 | 1 | 1 | N/A | N/A | -1 | 0 |
| Abuse - Shame | 1 | 1 | .01 | .01 | N/A | N/A | -1 | 0 |
| Abuse - Victimhood | 1 | 1 | .01 | .01 | N/A | N/A | -1 | 0 |
| Abuse - Other | 1 | 1 | .01 | .01 | N/A | N/A | -1 | 0 |
| Creation - Beliefs about Masculine Identities and Victimization | .67 | .67 | .67 | .80 | 0 | .33 | -.67 | .33 |
| Creation - LGBT | .50 | .50 | .50 | .67 | 0 | 0 | -.50 | .50 |
| Creation - Other | 1 | 1 | 1 | 1 | N/A | N/A | -1 | 0 |
| Creation – EPPM Efficacy of Recommended Response | 1 | 1 | 1 | 1 | N/A | N/A | -1 | 0 |
| Creation - EPPM -Recommended Response | .90 | .90 | .90 | .95 | 0 | .80 | -.90 | -.10 |
| Creation - EPPM - Self-efficacy | 1 | 1 | 1 | 1 | N/A | N/A | -1 | 0 |
| Creation – EPPM - Threat Severity | 1 | 1 | 1 | 1 | N/A | N/A | -1 | 0 |
| Creation – EPPM - Threat Susceptibility | 1 | 1 | 1 | 1 | N/A | N/A | -1 | 0 |
| Creation – Person - Celebrity | 1 | 1 | 1 | 1 | N/A | N/A | -1 | 0 |
| Creation – Person - Medic | 1 | 1 | 1 | 1 | N/A | N/A | -1 | 0 |
| Creation – Person - Other | 1 | 1 | 1 | 1 | N/A | N/A | -1 | 0 |
| Creation – Person - Regular Person | 1 | 1 | 1 | 1 | N/A | N/A | -1 | 0 |
| Creation – Appeal - Emotional | 1 | 1 | 1 | 1 | N/A | N/A | -1 | 0 |
| Creation - Appeal - Rational | 1 | 1 | 1 | 1 | N/A | N/A | -1 | 0 |
| Creation – Info - Isolation | 1 | 1 | 1 | 1 | N/A | N/A | -1 | 0 |
| Creation – Info - Legal | 1 | 1 | 1 | 1 | N/A | N/A | -1 | 0 |
| Creation – Info - Social Support | .92 | .92 | .92 | .96 | 0 | .83 | -.92 | .08 |
| Creation – Info - Types of Violence | 1 | 1 | 1 | 1 | N/A | N/A | -1 | 0 |
| Creation – Visual - Colour | 1 | 1 | 1 | 1 | N/A | N/A | -1 | 0 |
| Creation – Visual - Image | 1 | 1 | 1 | 1 | N/A | N/A | -1 | 0 |
| Creation – Visual - Text | 1 | 1 | 1 | 1 | N/A | N/A | -1 | 0 |
| Helplines - Beliefs | .67 | .67 | .56 | .75 | .5 | .33 | -.33 | 0 |
| Impressions – General - Negative | 1 | 1 | 1 | 1 | N/A | N/A | -1 | 0 |
| Impressions – General -Other | .83 | .83 | .83 | .91 | 0 | .67 | -.83 | .17 |
| Impressions – General -Positive | 1 | 1 | 1 | 1 | 1 | N/A | -1 | 0 |
| Impressions – Target -Negative | 1 | 1 | 1 | 1 | N/A | N/A | -1 | 0 |
| Impressions – Target -Other | .67 | .67 | .67 | .80 | 0 | .33 | -.67 | .33 |
| Impressions – Target -Positive | 1 | 1 | 1 | 1 | N/A | N/A | -1 | 0 |
| Impressions – Friends -Negative | 1 | 1 | 1 | 1 | N/A | N/A | -1 | 0 |
| Impressions – Friends -Other | 0 | 0 | 0 | 0 | 0 | -1 | 0 | 1 |
| Impressions – Friends -Positive | 1 | 1 | 1 | 1 | N/A | N/A | -1 | 0 |
| Impressions – Sweatshirt -Negative | .92 | .92 | .92 | .96 | 0 | .83 | -.92 | .08 |
| Impressions – Sweatshirt -Positive | 1 | 1 | 1 | 1 | N/A | N/A | -1 | 0 |
| Impressions – Xray - Negative | .86 | .86 | .86 | .92 | 0 | .71 | -.86 | -.14 |
| Impressions – Xray -Positive | 1 | 1 | 1 | 1 | N/A | N/A | -1 | 0 |
| Impressions - Identities | 1 | 1 | 1 | 1 | N/A | N/A | -1 | 0 |
| Impressions – EPPM -Efficacy of Response | 1 | 1 | 1 | 1 | N/A | N/A | -1 | 0 |
| Impressions – EPPM -Recommended Response | 1 | 1 | 1 | 1 | N/A | N/A | -1 | 0 |
| Impressions – EPPM - Self-Efficacy | 1 | 1 | 1 | 1 | N/A | N/A | -1 | 0 |
| Impressions – EPPM -Threat Severity | 1 | 1 | 1 | 1 | N/A | N/A | -1 | 0 |
| Impressions – EPPM -Threat Susceptibility | 1 | 1 | 1 | 1 | N/A | N/A | -1 | 0 |
| Impressions - Knowledge | 1 | 0 | 0 | 0 | 0 | -1 | 0 | 1 |
| Impressions - LGBT | 1 | 1 | 1 | 1 | N/A | N/A | -1 | 0 |
| Impressions - Other | .33 | .33 | .33 | .50 | 0 | -.33 | -.33 | .67 |
| Impressions - Perceived Effectiveness - Public | 1 | 1 | 1 | 1 | N/A | N/A | -1 | 0 |
| Impressions - Perceived Effectiveness - Target | 1 | 1 | 1 | 1 | N/A | N/A | -1 | 0 |
| Impressions – Person - Celebrity | 1 | 1 | 1 | 1 | N/A | N/A | -1 | 0 |
| Impressions – Person -Medic | 1 | 1 | 1 | 1 | N/A | N/A | -1 | 0 |
| Impressions – Person -Other | 0 | 0 | 0 | 0 | 0 | -1 | 0 | 1 |
| Impressions – Person -Regular Person | 1 | 1 | 1 | 1 | N/A | N/A | -1 | 0 |
| Impressions – Appeal - Emotional | 1 | 1 | 1 | 1 | N/A | N/A | -1 | 0 |
| Impressions - Type of Info - Isolation | 1 | 1 | 1 | 1 | N/A | N/A | -1 | 0 |
| Impressions - Type of Info - Types of Violence | 1 | 1 | 1 | 1 | N/A | N/A | -1 | 0 |
| Impressions - Type of Info - Social Processes | 1 | 1 | 1 | 1 | N/A | N/A | -1 | 0 |
| Impressions – Visual -Captivating | 1 | 1 | 1 | 1 | N/A | N/A | -1 | 0 |
| Impressions – Visual - Colour | 1 | 1 | 1 | 1 | N/A | N/A | -1 | 0 |
| Impressions – Visual- Composition | .77 | .77 | .77 | .87 | 0 | .54 | -.77 | .23 |
| Impressions – Visual -Text | .94 | .94 | .94 | .97 | 0 | .88 | -.94 | .06 |
| Knowledge – Beliefs about Masculinity | 1 | 1 | 1 | 1 | N/A | N/A | -1 | 0 |
| Knowledge – Beliefs about Society | 1 | 1 | 1 | 1 | N/A | N/A | -1 | 0 |
| Knowledge – Characterization - Channels | 1 | 1 | 1 | 1 | N/A | N/A | -1 | 0 |
| Knowledge – Characterization – Perceived Targets | 1 | 1 | 1 | 1 | N/A | N/A | -1 | 0 |
| Knowledge – Characterization - Positive | 1 | 1 | 1 | 1 | N/A | N/A | -1 | 0 |
| Knowledge – Characterization - Recollection | .88 | .88 | .88 | .93 | 0 | .75 | -.88 | -.13 |
| Knowledge – Characterization – Recommended Response | 1 | 1 | 1 | 1 | N/A | N/A | -1 | 0 |
| Knowledge – Characterization - Suggestions | 1 | 1 | 1 | 1 | N/A | N/A | -1 | 0 |
| Knowledge - Characterization - Target - Children | 1 | 1 | 1 | 1 | N/A | N/A | -1 | 0 |
| Knowledge - Characterization - Target - Men | 1 | 1 | 1 | 1 | N/A | N/A | -1 | 0 |
| Knowledge - Characterization - Target - Other | 1 | 1 | 1 | 1 | N/A | N/A | -1 | 0 |
| Knowledge - Characterization - Target - Women | 1 | 1 | 1 | 1 | N/A | N/A | -1 | 0 |
| Knowledge – Campaign Effects – General Population | .89 | .89 | .89 | .94 | 0 | .78 | -.89 | .11 |
| Knowledge - Campaign Effects - Interviewees | .86 | .86 | .86 | .92 | 0 | .71 | -.86 | .14 |
| Knowledge - Campaign Effects - Perceived Effect | .86 | .86 | .86 | .92 | 0 | .71 | -.86 | .14 |

**Appendix D**

*Characteristics of pictorial campaigns presented*

| Denomination used in this study | Promoting organisation | Year of release | Multi-channel (e.g. pictorial, video, audio) | Description |
| --- | --- | --- | --- | --- |
| “X-Ray” | APAV – Portuguese Association for Victim Support | 2016 | Yes, video format available | This campaign features a white background that has a presumed x-ray film on top of it. Inside this x-ray film that is deep black, at the top and centre a sentence states in blue bold font “This is the mark of a man who is a victim of domestic violence”. Bellow it there’s an x-ray of skull with a red spot/stain in it, close to the base of the skull. A white line points towards this red spot and presents the word “Shame”. At bottom of the campaign, in the white background, a sentence states "If you recognize it, call." Next to it the helpline logo (informing that the call is free and its working hours) as well as the logo for the promoting organisation are featured. |
| “Sweatshirt” | APAV – Portuguese Association for Victim Support | 2018 | Yes, video format available | This campaign has a grey background. At the top left corner, the logo for the entity responsible for its design is displayed. At the top centre the sentence “This sweatshirt may fit any person.” Is presented in black and bold, and below it, in smaller font, the sentence “Any person can be a victim of crime or of violence. If you are a victim of crime, contact APAV. Call 116 006.” Can be found. At the very centre of the image a grey sweatshirt is displayed unfolded, an on it, the word “Victim” is presented in red bold font. At the left lower end of the campaign the website, helpline logo (informing that the call is free and its working hours) as well as the logo for the promoting organisation are presented. |
| “Friends” | APAV – Portuguese Association for Victim Support | 2016 | No | Stylistically this campaign appears to be a direct shot from a camera without significant visual alterations. It portrays a “selfie” of a group of three people side by side (two men and one woman). The man on the left is smiling, and so is the woman who is in the middle, but the man on the right has a serious expression on his face. Additionally, this last man has a bruise on his left cheek. Each of the faces in the image are framed by a square with a corresponding name, simulating features found in social networks. The bruise in the man’s face is also framed by a square but does not have a description or name. On the top left of the campaign the website of the promoting organisation is presented, and on the bottom right it indicates that "If they leave a mark on you, you know with whom you can share". This message could be interpreted as a play on words given that the Portuguese expression used to convey that you could be the target of some kind of violence (i.e. “marcam”, to be the target of) is also used in Portuguese to describe mentions in social networks (i.e. tags in the English language). Below the promoting organisation logo and the helpline’s logo (informing that the call is free and its working hours) are featured. |
